# Hungarian Validation of the Individualized Neuromuscular Quality-of-Life Questionnaire (INQoL) in Adult Patients with Muscular Diseases

**DOI:** 10.3390/neurolint18050082

**Published:** 2026-04-28

**Authors:** Brigitta Ruszin-Perecz, Réka Héjas, Alexandra Makai, Nándor Hajdu, Dalma Jedlicska, Bence Ruszin-Perecz, Andrea Sipos, Endre Pál, Dávid Varga

**Affiliations:** 1 Department of Neurology, Medical School, University of Pécs, 7623 Pécs, Hungary; perecz.brigitta@pte.hu (B.R.-P.); hejas.reka@pte.hu (R.H.); ruszin.bence@pte.hu (B.R.-P.); sipos.andrea@aok.pte.hu (A.S.); varga.david@pte.hu (D.V.); 2 Faculty of Health Sciences, University of Pécs, 7621 Pécs, Hungary; alexandra.makai@etk.pte.hu; 3 Institute of Psychology, Eötvös Loránd University, 1053 Budapest, Hungary; hajdu.nandor93@gmail.com; 4 Department of Primary Health Care, University of Pécs, 7623 Pécs, Hungary; jedlicska.dalma@pte.hu; 5 Neuropathology Unit, Department of Pathology, University of Pécs, 7624 Pécs, Hungary

**Keywords:** muscular dystrophy, quality of life, validity, reliability

## Abstract

**Background/Objectives**: The Individualized Neuromuscular Quality-of-Life Questionnaire (INQoL) is a widely used measure of quality of life in patients with various neuromuscular diseases. This study aimed to adapt and test the validity and reliability of this measure in Hungarian patients with neuromuscular disease. Methods: According to the widely accepted method of validation, we first translated the original INQoL version into Hungarian, and then a native English speaker translated it back into English to test its validity. Following a pretest procedure, the INQoL was administered to 80 patients with various muscular diseases and 30 age-matched controls. The internal consistency and test–retest reliability were assessed. Concurrent validity was measured using the 36-item Short Form Survey (SF-36) questionnaire. Results: For all INQoL subscales, Cronbach’s alpha was above 0.7, demonstrating the reliability of the subscales. The highest Cronbach alpha value was for the Weakness subscale (0.983) and the lowest for the Treatment subscale (0.794). The intraclass correlation coefficient test values ranged from 0.810 (Treatment) to 0.988 (Pain), indicating excellent test–retest reliability. There was a strong correlation between the SF-36 Physical Function and multiple INQoL subscales, including Weakness (r = 0.754, *p* < 0.001), Fatigue (r = 0.704, *p* < 0.001), Activities (r = 0.744) *p* < 0.001, Independence (r = 0.791 *p* < 0.001), Body Image (r = 0.714 *p* < 0.001), and overall Quality of Life (r = 0.742 *p* < 0.001). Conclusions: Our findings indicate that the Hungarian-language adaptation of the questionnaire possesses adequate reliability and construct validity for assessing the quality of life in patients with muscular disorders.

## 1. Introduction

Muscle diseases comprise a heterogeneous group of disorders characterized by progressive muscle weakness and muscle wasting, leading to significant functional impairment. During the disease, patients often experience fatigue, psychological distress, reduced ability to perform daily activities, and impaired independence, all of which adversely affect their quality of life [1].

Owing to the clinical characteristics of these conditions and multisystem involvement in certain muscular dystrophies, such as myotonic dystrophy, Duchenne muscular dystrophy, and Becker muscular dystrophy, a comprehensive approach is required [2,3].

Because these are slowly deteriorating, long-standing diseases often accompanied by other non-muscle symptoms, patients frequently require healthcare services. Furthermore, due to the increasing number of symptoms and disease severity, hospital admissions have become frequent. For example, Howe et al. demonstrated that patients with myotonic dystrophy had increased healthcare resource utilization, substantially higher healthcare costs, and a high comorbidity burden compared with matched controls [4].

Quality of life (QoL) in neuromuscular diseases is influenced by a wide range of symptoms and complaints. Because most muscular disorders are currently incurable, improving patients’ QoL has become a therapeutic goal. Monitoring the QoL of individuals with muscular diseases can provide valuable information about the disease burden and, in the long term, help reduce its impact [5].

The study of QoL dates back to the eighteenth century, so its importance is well illustrated by the fact that researchers have been studying social well-being since the Age of Enlightenment. In today’s increasingly aging society, there is a growing emphasis on improving the quality of life of patients with chronic diseases [6].

One of the most important therapeutic goals for people with chronic infirmity is to improve their health and well-being. Thus, in addition to the general quality-of-life assessment, Health-Related Quality of Life (HRQoL), which is measured using self-administered questionnaires, plays an important role. General health-related quality-of-life questionnaires, such as the EQ-5D [7], are among the most commonly used instruments for this purpose. Another widely applied generic measure is the Short Form-36 (SF-36), which has also been validated in the Hungarian population [8,9,10]. Disease-specific quality-of-life questionnaires have been developed to measure disease-related symptoms more accurately. Neuromuscular diseases represent an important group of chronic conditions, with a focus on muscle weakness and pain. Currently, there is no disease-specific questionnaire for muscle disorders available in the Hungarian language to measure quality of life. It is assumed that if the quality of life of patients with muscular disorders is improved, the use of hospital resources can be reduced. Furthermore, we can obtain a complete picture of the condition of patients with muscular diseases [5].

In our study, we used the “Individualized Neuromuscular Quality of Life” (INQoL) questionnaire to score the QoL in patients with muscular diseases. It was developed in 2007 [11]. Later, the questionnaire was translated and validated for the Italian, Serbian, Spanish, Japanese, Korean, and Dutch populations, as well as in the USA [12,13,14,15,16,17,18].

## 2. Materials and Methods

### 2.1. Measures

INQoL consists of 45 questions grouped into 11 subscales across three dimensions: symptoms, life domains, and treatment effects. The symptoms dimension includes seven subscales assessing neuromuscular symptoms (weakness, locking, pain, fatigue, droopy eyelids, double vision, and swallowing difficulties); the life domains dimension includes five subscales evaluating the impact of muscle disease on patients’ lives (activities, independence, relationships, emotions, and body image); and the treatment effects dimension includes two subscales assessing experienced and expected treatment effects.

The items are assessed using a 7-point Likert scale, reflecting the degree of impact of specific symptoms or muscle disease on different aspects of the patient’s life. Subscale scores indicate the degree of muscle disease symptoms or life domains. Subscale scores were calculated by averaging item responses within each subscale and subsequently transforming them to a 0–100 scale, with higher scores indicating greater disease impact and poorer quality of life. The overall QoL score is calculated from the ‘life domains’ dimension, five subscales assessing the influence of muscle disease on particular areas of life, and reflects the patients’ overall HRQoL [19]. The questionnaire is based on the patient’s subjective complaints and judgment; therefore, it seems to be useful for measuring QoL [11].

For external validation of the questionnaire, we used the 36-item Short-Form Questionnaire-36 (SF-36), a general quality-of-life questionnaire widely used to assess patients [9]. The SF-36 is a commonly used, generic, health-related quality-of-life questionnaire. It assesses health status across eight domains, including physical, mental, and social functioning. The SF-36 allows comparison of quality of life across different diseases and populations; however, as a generic instrument, it may be less sensitive to disease-specific issues in neuromuscular disorders [9].

### 2.2. Translation and Cultural Adaptation

INQoL was translated and cross-culturally adapted following a standard, previously used methodology [19]. Prior to initiating the validation study, written permission was obtained from the original developers of this questionnaire. With their permission, two independent individuals translated the questionnaire from English into Hungarian. A combined version of the two Hungarian translations was prepared, and a third person translated this combined version from Hungarian into English. No significant differences were found between the original English questionnaire and the text.

The Hungarian version was pretested on 17 healthy participants. Based on their recommendations, we changed some words and phrases in the questionnaire to make it more understandable and avoid mistakes that might be the consequence of a misunderstanding. Thus, in question No. 3, the word “considerable” was changed to “significant,” and in question No. 7, the word “connection” was used instead of “relation.” Based on the observations of the pre-testers, in the first question, we changed the instructions from “go to question 2A” to “go to question 2” if the answer is no. The final questionnaire was completed by the patients.

### 2.3. Participants

Patients were recruited from the Neuromuscular Unit, Department of Neurology, University of Pécs, Hungary. The inclusion criteria for participation in this survey were (1) a confirmed diagnosis of a muscular disorder, (2) age ≥ 18 years, and (3) the ability to read and write adequately if patients did not have significant cognitive decline, as judged by an investigator during history-taking. Participants were excluded if their diagnosis could not be reliably confirmed during the study period or if they refused to complete the questionnaire.

Eighty patients with different types of muscular diseases participated in this study. Most patients had myotonic dystrophy type 1 (DM1; *n* = 47). Other diseases included myopathy with proximal weakness (*n* = 10), mitochondrial myopathy (MTD, *n* = 6), motor neuron disease (MND; *n* = 2), myasthenia gravis (MG; *n* = 2), limb girdle muscular dystrophy (LGMD; *n* = 3), facioscapulohumeral–humeral muscular dystrophy (FSHD; *n* = 5), Becker muscular dystrophy (BMD; *n* = 2), and spinal muscular atrophy (SMA, *n* = 3). Thirty healthy subjects were included as the age- and sex-matched control group, who did not have any significant diseases in their medical history.

After completing the Hungarian version of the INQoL, all participants completed the SF-36 survey on the same day.

### 2.4. Ethical Considerations

This study was approved by the Institutional Review Board (IRB) of the University of Pécs, Hungary (approval number: 5505/2014). Written informed consent was obtained from all patients according to the IRB protocol.

### 2.5. Statistical Analysis

Reliability was evaluated using Cronbach’s alpha values and a test–retest analysis. To assess the internal consistency of the questionnaire, Cronbach’s alpha was calculated. Values of ≥0.70 were considered acceptable, ≥0.80 good, and ≥0.90 excellent [20]. Test–retest reliability was evaluated using the intraclass correlation coefficient (ICC). Based on established guidelines, ICC values below 0.50 were considered poor, between 0.50 and 0.75 moderate, between 0.75 and 0.90 good, and above 0.90 excellent reliability [21]. We conducted a test–retest study with 17 patients in which the test was performed again. Validity was assessed using Spearman’s rank correlation. Convergent validity was evaluated by comparing the subscale scores of the INQoL with the corresponding subscales of the previously validated generic quality-of-life questionnaire, the SF-36. The Mann–Whitney test was used to test the discriminant validity of the measurement tools and to compare INQoL subscale scores across different diagnostic subgroups (between patients with muscle disease and healthy controls). Further analyses were conducted using the Mann–Whitney U test to examine the differences between demographic subgroups, and Spearman’s rank correlation was applied to measure the relationship between INQoL scores and age. Differences were considered statistically significant at *p* ≤ 0.05. IBM SPSS software (version 25, IBM Corporation, Armonk, NY, USA) was used for statistical analyses.

## 3. Results

This study included 45 male and 65 female patients with an average age of 47.07 years (SD ± 14.03 years). The demographics of the study participants are presented in Table 1.

Details on within-group variance across subscales are presented in Supplementary Table S1.

### 3.1. Cronbach’s Alpha and Test–Retest Reliability

For all subscales, Cronbach’s alpha was above 0.7, demonstrating the reliability of the subscales. The highest value was for the Weakness subscale (0.983), and the lowest was for the Treatment subscale (0.794). The data are presented in Table 2. Test–retest reliability was assessed using the intraclass correlation coefficient (ICC). The ICC test results confirmed the conclusions drawn from Cronbach’s alpha test. A strong correlation was found between the test–retest scores for the tested subscales. ICC values ranged from 0.810 (95% CI (0.273–0.987)) (Treatment) to 0.988 (95% CI (0.973–0.996)) (Pain), indicating excellent test–retest reliability. All subscales had ICC values above 0.8, and most had values above 0.9, indicating particularly strong reliability.

Examining the subscales, our results showed a close correlation between the test–retest values (Table 3).

### 3.2. Convergent Validity with SF-36

During the convergent validity study, we compared the results of the subscales of the SF-36 questionnaire with the values of the INQoL subscales. Based on our results, the two questionnaires correlated well with each other, and there was a strong significant relationship between the two continuous variables. It should be mentioned that there is a strong correlation between the SF-36 Physical Function and multiple INQoL subscales, including Weakness (R = 0.754), Fatigue (R = 0.704), Activities (R = 0.744), Independence (R = 0.791), Body Image (R = 0.714), and overall Quality of Life (R = 0.742).

A moderate but statistically significant correlation was observed between the subscales of the SF-36 questionnaire, which tested the emotional life and the scores of the Social Relationships, Emotions, and Body Image subunits of the INQoL questionnaire. The SF-36 emotional wellness subscale showed a poor correlation with the INQoL Activities and Independence scores. Finally, compared with the others, the SF-36 social functioning variable did not show a significant correlation with the INQoL Weakness, Fatigue and Activities variables (Table 4).

Between-group comparisons demonstrated highly significant differences across all primary INQoL domains (all *p* < 0.001), including the physical, functional, and psychosocial components. These results indicate the strong discriminative capacity of the instrument. Although treatment-related domains also reached statistical significance (perceived treatment effects: *p* = 0.019; expected treatment effects: *p* = 0.014), the magnitude of these differences appeared less pronounced.

### 3.3. Discriminate Validity of the INQoL Questionnaire

Healthy controls showed minimal impact across all INQoL domains, with mean scores close to zero (e.g., weakness: mean = 0.00, SD = 0.00; fatigue: mean = 1.93, SD = 6.26; QoL: mean = 1.06, SD = 2.17). In contrast, the DM1 group demonstrated markedly higher scores, particularly in weakness (mean = 51.96, SD = 33.50), fatigue (mean = 42.89, SD = 34.65), activities (mean = 42.62, SD = 30.88), and overall QoL (mean = 32.96, SD = 23.85). Similarly, the other neuromuscular disorder group showed elevated scores across domains, with weakness (mean = 55.98, SD = 33.33), fatigue (mean = 48.64, SD = 28.19), activities (mean = 48.52, SD = 25.18), and QoL (mean = 39.90, SD = 21.46) being the most affected. The three subgroups showed significantly different subscores in each domain (Table 5).

### 3.4. Demographic and Diagnosis-Specific Correlations

Analysis of demographic variables (gender and age) and INQoL scores revealed no significant correlations (*p* > 0.05). Although the patients belonged to different subgroups according to the type of myopathy, there was no significant difference in the subscale scores between the subgroups regarding the subscale scores. When analyzed by diagnosis, patients with DM1 and other muscle diseases had significantly higher scores on all INQoL subscales (except for Treatment) than healthy controls, indicating a significant reduction in quality of life in these patients.

## 4. Discussion

In this study, we validated the Hungarian version of the Individualized Neuromuscular Quality-of-Life Questionnaire. During the validation process, we demonstrated that the Hungarian version of the questionnaire is a reliable and valid instrument for measuring the quality of life in patients with muscular disorders.

Psychometric assessment revealed excellent internal consistency for all the subscales. The Cronbach’s alpha values (0.794–0.983) were similar to those obtained in other language validation studies, for example, the original English (0.76–0.96), Italian (0.81–0.96), Serbian (0.85–0.97), Korean (0.81–0.99) and Japanese (0.84–0.96) versions [11,12,13,14]. This suggests that the Hungarian translation maintained the reliability of the original questionnaire [11]. Test–retest reliability analysis demonstrated the stability of the INQoL subscales over time. The ICC values for all subscales were excellent (0.810–0.988), indicating that the constructs measured by the questionnaire were stable over time and that the measurement was repeatable. These results are consistent with those of previous validation studies, which also showed good test–retest reliability [11,12,13,14].

When examining the convergent validity of the INQoL questionnaire, we found significant correlations with the corresponding subscales of the SF-36, specifically between the SF-36 Physical Function subscale and the INQoL subscales measuring physical aspects (Weakness, Fatigue, Activities, Independence). This result confirms that the Hungarian version of the INQoL is a valid measure of the physical effects of neuromuscular diseases. However, the more moderate correlation between the subscales measuring emotional and social aspects may suggest that the INQoL approaches these domains from a different perspective than the SF-36 does. These findings suggest that the INQoL may provide a more specific assessment of quality-of-life in patients with muscular diseases than generic quality of life instruments such as the SF-36.

One important finding of our study was that we found no significant correlation between INQoL scores and sex or age. This suggests that the questionnaire is suitable for assessing the quality of life of patients of different genders and ages and that scores are primarily influenced by the nature and severity of the disease rather than by demographic factors.

When comparing patients with different muscle diseases, no significant differences were found between the disease groups in the INQoL subscales. This may suggest that different neuromuscular diseases affect the quality of life in a similar way, and the lack of significant differences indicates that the questionnaire reflects the overall disease burden independent of myopathy subtype. However, a larger sample size in each disease group would be needed to assess this more accurately. Comparisons between patients with muscle disease (DM1 and other muscle diseases) and healthy controls clearly showed a significant reduction in QoL in both disease groups compared to healthy controls. This confirms that the INQoL questionnaire can differentiate between healthy and diseased populations and is a sensitive measure of the impact of muscle diseases on QoL [11].

The results obtained for myotonic dystrophy (DM1) are in line with previous studies [13,14], which also showed a significant reduction in the quality of life in this patient group. The multisystemic nature of DM1, affecting the musculoskeletal system, heart, lungs, endocrine glands, central nervous system, and other organ systems, may explain the extensive deterioration in QoL [2,13,14].

A limitation of our study is the small sample size, especially in certain muscle disease subgroups, and the heterogeneity of the study population. Further research with larger and more homogeneous patient groups is needed to accurately assess the applicability of the questionnaire on specific muscle diseases. Some diagnostic subgroups had small sample sizes (e.g., MND, MG, and BMD), which may limit the stability of subgroup comparisons and reduce statistical power. While overall reliability measures (Cronbach’s alpha and ICC) were strong, these results primarily reflect the total sample rather than individual diagnostic subgroups.

Overall, the validation of the Hungarian version of the INQoL questionnaire will contribute to a better understanding and assessment of the quality of life of patients with neuromuscular diseases and can facilitate the development of personalized therapeutic approaches aimed at improving patients’ quality of life.

Notably, the INQoL Treatment subscale showed the lowest Cronbach’s alpha (0.794) and ICC (0.810), although these were still within an acceptable range. This is in line with other validation studies and may suggest that treatment experience and expectations may be more variable than other aspects of QoL [11,12,13,14].

## 5. Conclusions

Based on the psychometric properties of the Hungarian version of the INQoL questionnaire, it can be concluded that it is a reliable and valid instrument for measuring the quality of life of patients with neuromuscular disease. Evidence of excellent internal consistency, good test–retest reliability, and convergent validity with the SF-36 questionnaire supports the applicability of the Hungarian version of the questionnaire.

A comparison of patients with different muscle diseases and healthy controls confirmed that the INQoL questionnaire is a sensitive measure of the impact of muscle disease on QoL. Therefore, the questionnaire is suitable for assessing the quality-of-life of individual patients in clinical practice and for evaluating the effectiveness of therapeutic interventions. [11] The Hungarian version of the INQoL questionnaire could be a valuable tool in clinical research, especially in studies measuring the impact of new therapies on QoL. The questionnaire is specifically focused on the characteristics of neuromuscular diseases and can therefore measure changes in the quality of life in these diseases more sensitively than general quality of life questionnaires [12,13,14].

## Figures and Tables

**Table 1:** Demographics of the study participants.

	Patients (*n* = 80)	Controls (*n* = 30)
Age (years, mean ± SD)	47.33 ± 15.01	47.53 ± 14.57
Age (years, min–max)	30–56	18–83
Sex (males)	*n* = 31 (40.26%)	*n* = 11 (36.77%)

**Table 2:** The results of Cronbach-alpha test.

Subscales	Cronbach Alpha
Weakness score	0.983
Muscle ‘locking’ score	0.945
Pain	0.968
Fatigue	0.964
Activities score	0.928
Independence score	0.962
Social relationships	0.951
Emotions score	0.919
Body Image score	0.938
QoL	0.963
Treatment	0.794

All subscales showed Cronbach’s alpha values above 0.70, supporting the internal consistency of the questionnaire and the reliability of the subscales. This was also supported by the results of the intraclass correlation coefficient (ICC) tests.

**Table 3:** Intraclass Correlation Coefficients for Test–Retest Reliability.

Variables Analyzed	Intraclass Correlation Coefficient	95% Confidence Interval	
		Lower Bound	Upper Bound
Weakness score	0.978	0.955	0.992
Muscle ‘locking’ score	0.971	0.938	0.989
Pain	0.988	0.973	0.996
Fatigue	0.981	0.961	0.993
Activities score	0.963	0.917	0.989
Independence score	0.985	0.969	0.994
Social relationships	0.942	0.878	0.982
Emotions score	0.931	0.870	0.971
Body Image score	0.973	0.949	0.988
QoL	0.962	0.927	0.985
Treatment	0.810	0.273	0.987

**Table 4:** Convergent validation of the INQoL and SF-36.

SF-36 INQoL	Physial Function	PHYSICAL health	Emotions	Energy	Emotional Wellness	Social Functioning	Pain	General Health	Health Change
Weakness score	−0.754 **	−0.523 **	−0.354 **	−0.446 **	−0.267 *	−0.237	−0.345 **	−0.673 **	−0.466 **
Muscle ‘locking’ score	−0.517 **	−0.454 **	−0.350 **	−0.353 **	−0.455 **	−0.377 **	−0.570 **	−0.553 **	−0.345 **
Pain	−0.459 **	−0.465 **	−0.318 *	−0.319 *	−0.263 *	−0.273 *	−0.606 **	−0.494 **	−0.427 **
Fatigue	−0.704 **	−0.539 **	−0.492 **	−0.546 **	−0.326 *	−0.208	−0.448 **	−0.660 **	−0.567 **
Activities score	−0.744 **	−0.552 **	−0.324 *	−0.414 **	−0.256	−0.203	−0.360 **	−0.586 **	−0.428 **
Independence score	−0.791 **	−0.525 **	−0.355 **	−0.434 **	−0.216	−0.335 *	−0.449 **	−0.655 **	−0.437 **
Social relationships	−0.699 **	−0.482 **	−0.211	−0.383 **	−0.294 *	−0.360 **	−0.400 **	−0.587 **	−0.424 **
Emotions score	−0.448 **	−0.402 **	−0.258	−0.500 **	−0.489 **	−0.397 **	−0.347 **	−0.539 **	−0.337 **
Body Image score	−0.714 **	−0.392 **	−0.220	−0.437 **	**−0.228**	−0.316 *	−0.569 **	−0.634 **	−0.535 **
QoL	−0.742 **	−0.511 **	−0.318 *	−0.506 **	−0.330 *	−0.402 **	−0.467 **	−0.678 **	−0.459 **

Data show Spearman’s correlation factor (R) among SF-36 and InQoL subscale values, significant values marked by * (<0.05) or ** (<0.001).

**Table 5:** Discriminate validity of the INQoL questionnaire.

		Weakness Score	Muscle ‘Locking’ Score	Pain	Fatigue	Activities Score	Independence Score	Social Relationships	Emotions Score	Body Image Score	QoL	Perceived Treatment Effects	Expected Treatment Effects
Healthy	Mean	0.00	0.00	0.53	1.93	5.45	0.74	0.00	0.65	2.04	1.06	0.00	0.00
	SD	0.00	0.00	2.88	6.26	10.95	2.82	0.00	3.07	4.67	2.17	0.00	0.00
DM1	Mean	51.96	37.96	37.18	42.89	42.62	38.06	22.01	25.35	38.18	32.96	13.83	12.77
	SD	33.50	34.11	33.99	34.65	30.88	33.09	19.81	20.34	32.24	23.85	26.08	24.75
Other *p*	Mean	55.98	32.85	36.20	48.64	48.52	42.26	27.02	33.33	41.33	39.90	13.38	16.92
	SD	33.33	28.47	26.44	28.19	25.18	31.45	21.90	22.72	28.77	21.46	27.40	32.11
		<0.001	<0.001	<0.001	<0.001	<0.001	<0.001	<0.001	<0.001	<0.001	<0.001	0.019	0.014
Z		−6.129	−5.478	−5.189	−5.443	−5.156	−5.300	−6.648	−6.366	−5.250	−6.244	−2.833	−2.833
Effect size		0.70	0.62	0.59	0.62	0.59	0.60	0.76	0.73	0.60	0.71	0.32	0.32

Data show mean and standard deviation (SD) of subscores in healthy and patient groups. The difference was significant in each subdomain.

## Data Availability

Data are available at the correspondent author upon request.

## Supplementary Materials

The following supporting information can be downloaded at https://www.mdpi.com/article/10.3390/neurolint18050082/s1, Table S1: Within-group variance across subscales.

## Abbreviations

The following abbreviations are used in this manuscript.

DM1	myotonic dystrophy type 1
FSHD	Facioscapulohumeral dystrophy
HRQoL	Health-related quality of life
ICC	intraclass correlation coefficient
INQoL	Individualized Neuromuscular Quality-of-Life Questionnaire
LGMD	Limb-girdle muscular dystrophy
MG	myasthenia gravis
MND	motor neuron disease
SF-36	Short-form Questionnaire 36
SMA	spinal muscular atrophy
QoL	Quality of life
