# Supplementary material for: Hungarian Validation of the Individualized Neuromuscular Quality-of-Life Questionnaire (INQoL) in Adult Patients with Muscular Diseases
Source: Neurol Int. 2026 Apr 28;18(5):82. doi: 10.3390/neurolint18050082 (PMC13210065; doi:10.3390/neurolint18050082)
Supplement: Supplementary file 1 [file neurolint-18-00082-s001.zip › neurolint-4200355-supplementary.pdf]

Table S1.Within group variance across subscales

		Weakness score	Muscle 'locking' score	Activities score	Independence score	Social relationships	Emotions score	Body Image score	Qol	Perceived Treatment effects	Expected treatment effects
N	Valid	110,00	110,00	110,00	110,00	110,00	110,00	110,00	110,00	110,00	110,00
Mean		39,00	26,08	34,25	29,14	17,51	21,01	29,27	26,34	9,92	10,53
Std. Deviation		37,12	31,48	30,67	32,60	20,66	22,29	31,14	25,04	23,34	24,62
Minimum		0,00	0,00	0,00	0,00	0,00	0,00	0,00	0,00	0,00	0,00
Maximum		100,00	94,74	97,33	100,00	93,52	80,56	91,67	84,44	83,33	100,00
